# SpliceMutr Enables Pan-Cancer Analysis of Splicing-Derived Neoantigen Burden in Tumors

**DOI:** 10.1158/2767-9764.CRC-23-0309

**Published:** 2024-12-16

**Authors:** Theron Palmer, Michael D. Kessler, Xiaoshan M. Shao, Archana Balan, Mark Yarchoan, Neeha Zaidi, Tamara Y. Lopez-Vidal, Ali M. Saeed, Jessica Gore, Nilofer S. Azad, Elizabeth M. Jaffee, Alexander V. Favorov, Valsamo Anagnostou, Rachel Karchin, Daria A. Gaykalova, Elana J. Fertig, Ludmila Danilova

**Affiliations:** 1 Department of Biomedical Engineering, Johns Hopkins University, Baltimore, Maryland.; 2 Convergence Institute, Johns Hopkins University, Baltimore, Maryland.; 3 Department of Oncology, Sidney Kimmel Comprehensive Cancer Center, Johns Hopkins University, Baltimore, Maryland.; 4 Bloomberg-Kimmel Institute for Cancer Immunotherapy, Sidney Kimmel Comprehensive Cancer Center, Johns Hopkins University, Baltimore, Maryland.; 5 Biochemistry, Cellular and Molecular Biology (BCMB) Graduate Program, Johns Hopkins University School of Medicine, Baltimore, Maryland.; 6 Laboratory of Systems Biology and Computational Genetics, Vavilov Institute of General Genetics, Moscow, Russia.; 7 Department of Otorhinolaryngology-Head and Neck Surgery, Marlene and Stewart Greenebaum Comprehensive Cancer Center, University of Maryland School of Medicine, Baltimore, Maryland.; 8 Institute for Genome Sciences, University of Maryland School of Medicine, Baltimore, Maryland.; 9 Department of Applied Mathematics and Statistics, Johns Hopkins University, Baltimore, Maryland.

## Abstract

Aberrant alternative splicing can generate neoantigens, which can themselves stimulate immune responses and surveillance. Previous methods for quantifying splicing-derived neoantigens are limited by independent references and potential batch effects. Here, we introduce SpliceMutr, a bioinformatics approach and pipeline for identifying splicing-derived neoantigens from tumor and normal data. SpliceMutr facilitates the identification of tumor-specific antigenic splice variants, predicts MHC-binding affinity, and estimates splicing antigenicity scores per gene. By applying this tool to transcriptomic data from The Cancer Genome Atlas, we generate splicing-derived neoantigens and neoantigenicity scores per sample and across all cancer types and find numerous correlations between splicing antigenicity and well-established biomarkers of antitumor immunity. Notably, carriers of mutations within splicing machinery genes have higher splicing antigenicity, which provides support for our approach. Further analysis of splicing antigenicity in cohorts of patients with melanoma treated with mono- or combined immune checkpoint inhibition suggests that the abundance of splicing antigens is reduced post-treatment from baseline in patients who progress. We also observe increased splicing antigenicity in responders to immunotherapy, which may relate to an increased capacity to mount an immune response to splicing-derived antigens. We find the splicing antigenicity to be higher in tumor samples when compared with normal, that mutations in the splicing machinery result in increased splicing antigenicity in some cancers, and higher splicing antigenicity is associated with positive response to immune checkpoint inhibitor therapies. Furthermore, this new computational pipeline provides novel analytical capabilities for splicing antigenicity and is openly available for further immuno-oncology analysis.

**Significance:**

SpliceMutr shows that splicing antigenicity changes in response to ICI therapies and that native modulation of the splicing machinery through mutations increases the contribution of splicing to the neoantigen load of some The Cancer Genome Atlas cancer subtypes. Future studies of the relationship between splicing antigenicity and immune checkpoint inhibitor response pan-cancer are essential to establish the interplay between antigen heterogeneity and immunotherapy regimen on patient response.

## Introduction

Tumor immune surveillance of premalignant or cancerous lesions is contingent on the recognition of tumor-specific neoantigens presented on the surface of tumor cells and their elimination by the immune system. Despite the continuous pressure by the immune system to prevent the growth of nascent tumor clones, cancer cells evolve mechanisms of immune suppression that prevent their effective elimination and enable their escape and growth. Upregulation of immune checkpoints, such as CTLA-4, by regulatory T cells, Tregs, or PD-1/PD-L1 axis in the tumor microenvironment results in abrogation of effector T-cell function that compromises immune clearance of tumor clones. This sets the basis for the clinical development of immune checkpoint inhibitors that remove the brakes of these inhibitory checkpoints and reinvigorate antitumor immunity (1, 2). Identifying biomarkers and functional determinants of response to ICIs is an active area of research. Tumor mutational burden (TMB) has been widely recognized as a biomarker of response to immunotherapy, as a high mutational burden is thought to give rise to more neoantigens that can be recognized and eliminated by immune cells (3). However, TMB is not a universal biomarker of immune checkpoint inhibition (ICI) response or cytotoxic T-cell infiltration (4, 5). Although mutations represent one source of alteration to the protein structure of genes in cancer, further transcriptional and translational modifications to protein structure can also induce tumor antigens independent of mutation status if these modifications introduce altered peptide sequences to the protein. Identifying these mutation-independent tumor antigens is key to mapping the determinants of immunologic tumor control and subsequent clinical responses.

Currently, several computational algorithms predict immunogenic neoantigens based on the extraction of various features, including expression levels of mutant alleles, MHC presentation, mutant epitope-MHC binding affinity, and probability of being recognized by the T cells. These algorithms focused on tumor-specific nonsynonymous variants, including indels. However, given the incomplete ability of TMB and canonical predicted neoantigens to accurately stratify patients based on response to ICI therapies, alternate sources of neoantigen production and methodologies to identify them are needed.

Abnormal RNA splicing in cancer cells contributes to many oncogenic processes, and splicing modulators have emerged as attractive targets for cancer therapy (6–10). The association between splicing dysregulation and immune recognition across cancers has been explored in previous analyses (5, 10–12). Notably, previous work showed that in breast and ovarian cancers, the number of MHC-bound peptides derived from tumor-specific splicing events was higher, on average, than those resulting from single nucleotide variants (11). The capacity of the splicing events to create splicing-impacted peptides that can bind to the MHC complex suggests that they may have translational implications on the outcome of immunotherapy treatment. For example, a study evaluating the anticancer drug indisulam, an RBM39 inhibitor that disrupts RNA splicing, in a mouse model of melanoma found that MHC-I presentation of splicing-derived immunogenic peptides induced T-cell activation and amplified the therapeutic efficacy of PD-1 inhibition (13). Despite mounting evidence that dysregulated splicing represents a source of viable tumor neoantigens, their functional relevance and clinical significance in the context of immunotherapy responses remain poorly explored and require in-depth studies.

Most current computational pipelines for neoantigen prediction do not consider splicing-derived neoantigens and thus overlook a potentially large fraction of the neoantigen repertoire that may be informative of antitumor immune responses. Recently, several tools to infer antigens from splice variants have emerged in the literature (5, 14, 15). This requires limiting analysis to tumor-specific splice variants prior to predicting their antigenicity from sequence. Many current methodologies for splicing neoantigen analysis using short-read RNA sequencing (RNA-seq) data depend upon splice graph augmentation (11, 16) or splice junction–centric outlier analysis using mixed-tissue references (15). These methods often rely on tissue-wide references from cohorts other than the reference RNA-seq datasets used to measure splicing in tumors, potentially introducing batch effects. These limitations can be overcome through analysis approaches comparing tumor-specific splice antigens to those observed in normals with differential splicing analysis using tools like LeafCutter (17), SEVA (18), or LeafCutterMD (19).

In the current study, we have developed a methodology based on splice junction–centric splicing analysis, SpliceMutr, that calculates tumor and normal aberrant splicing-derived neoantigen load per gene and per sample that we use to compare splicing antigenicity to current measures of the immune response. Our pipeline enables a unique evaluation of splicing-derived antigens using statistically rigorous differential or outlier splicing analysis for the identification of target splice junctions between samples sequenced using the same sequencing technology and from the same tissue of origin. SpliceMutr modifies the reference transcriptome using these identified splice junctions, filters the reference transcriptome based on the calculated coding potential, and generates candidate peptide kmers using the altered reference transcriptome that are then tested for MHC peptide–binding potential. SpliceMutr then uses these candidate peptides for the calculation of the splicing antigenicity score, which accounts for the tumor purity of the sample, length of the peptide derived from the splice junction–modified transcript, and the expression of the splice junction. We apply SpliceMutr to The Cancer Genome Atlas (TCGA) dataset to define the interplay between the splicing antigenicity and TMB. We also checked the role mutations in the splicing factor machinery play in the level of splicing antigenicity. Finally, we used a cohort of ICI-treated patients with melanoma to define how splicing antigenicity relates to clinical outcomes with immune checkpoint blockade. Altogether, our analyses with SpliceMutr lead us to hypothesize that the diversity of neoantigens resulting from the tumor splicing antigenicity increases the ability of the immune system to mount a successful immune response, which warrants future mechanistic studies.

## Materials and Methods

### Preprocessing RNA-seq data

SpliceMutr requires splicing-aware aligned RNA-seq data as an input. In this study, all data were aligned using STAR version 2.7.3a using the basic two pass mode and six threads (20). Both the .bam and the SJ.out.tab files are used moving forward in the analysis. Each .bam file is used for sample genotyping by arcasHLA (21). The SJ.out.tab file is a file containing a set of high-confidence splice junctions output by the STAR aligner. This SJ.out.tab file is first filtered to contain only canonical splice motifs, and then the data from the SJ.out.tab file is transferred to a .junc file format that is readable by LeafCutter (17) and LeafCutterMD (19) using custom scripts available from https://github.com/FertigLab/splicemutr or via zenodo (doi: 10.5281/zenodo.11116273).

### Running LeafCutter

Each .junc file and the metadata necessary to run LeafCutter (17) differential junction usage analysis are created using custom scripts and by hand. Initially, LeafCutter creates a set of clusters of splice junctions that overlap based on their coordinates and the existence of at least three reads, providing evidence of the existence of the splice junction in the data. The script that performs this analysis is modified to accept the non-BED formatted .junc files created in the preprocessing stage. After clustering this way, we perform differential splice junction usage analysis using LeafCutter with at least six samples per comparison.

### Running LeafCutterMD

LeafCutterMD (19) is an algorithm based on LeafCutter that evaluates outlier usage of splice junctions in a one-versus-many manner. Splice junction clustering is done exactly the same as it is when running LeafCutter differential splice junction usage analysis. The difference when running LeafCutterMD is the script that processes the output of this clustering result as well as the metadata generated to perform the analysis. The outlier analysis is done in a one-versus-many fashion, so the files that determine sample groupings contain a single sample belonging to the target group and then a larger number of samples belonging to the comparator group. The splice junctions identified as having outlier expression are those of the target group having outlier usage when compared with the appropriate splice junction cluster of the comparator group. Creating the many target and comparator groups to be run by LeafCutterMD, as well as collapsing the many one-versus-many outlier analysis done by LeafCutter, is carried out using custom analysis scripts.

### Sample genotyping arcasHLA

Sample genotyping using arcasHLA is carried out by first running the extract functionality of arcasHLA on the STAR alignment output BAM files and then running the genotype functionality of arcasHLA on the output from the extract. The extract functionality of the arcasHLA toolset extracts reads that map to chromosome 6 of the human genome. The genotype functionality of arcasHLA gives the genotype of the sample given the reads mapped to chromosome 6 of the alignment file. The genotype per sample is then extracted and saved for per sample MHC:peptide binding affinity predictions while running that portion of SpliceMutr.

### Running MHCnuggets

The per-sample genotype output from arcasHLA as well as the tumor-specific and normal-specific kmers output from SpliceMutr, are input into MHCnuggets (22). Each kmer undergoes binding affinity prediction using each MHC class 1 allele from each sample-specific genotype to determine a set of tumor-specific and normal-specific antigenic kmers.

### The LGC (open reading frame Length and GC content) coding potential calculation

Briefly, the modified LGC coding potential is calculated as follows:

$$P={\left(1-f\right)}^{n-1}\cdot f,$$ (A)

$$f\;=\;a_{0}+a_{1}P_{GC}+a_{2}P_{GC}^{2\;}+a_{3}P_{GC}^{3},$$ (B)

$$P_{GC}=P_{C}+P_{G},$$ (C)

where $P_{C}$ is the probability of cytosine, $P_{G}$ is the probability of guanine, P is the probability of the open reading frame (ORF) in the sequence, f is the probability of finding a stop codon in the sequence, n is the number of sense codons in the sequence, and the parameters $a_{0}$ through $a_{3}$ are coefficients learned from protein-coding human transcripts or long noncoding human transcripts. This coding potential can then be used to calculate the differential coding potential (*L*) between the coding LGC score (*P*_*c*_) and the long noncoding LGC score (*P*_*nc*_) for the same transcript:

$$L\;=\;\log_{2}\;\left(\frac{P_{c}}{P_{nc}}\right).$$ (D)

### Calculating the pseudo purity

$$Pseudo\;purity=1-\frac{ESTIMATE}{\max\;\left(ESTIMATE\right)\;+\;\left(\max\;\left(ESTIMATE\right)\;\times \;2.220446E-16\right)}.$$ (E)

The pseudo purity is calculated for a cohort using the ESTIMATE (23) score normalized by the maximum ESTIMATE score for the cohort plus the product of the maximum and machine epsilon in the R statistical package. This is so that the maximum ESTIMATE score for the cohort does not result in a sample with a tumor purity of zero. Only samples with tumor purity greater than 0.01 are used for analysis.

### Calculating the differential agretopicity

$$DA\;\left(G\right)\;=\;\;\log_{2}\;\left(SA_{T}/SA_{N}\right).$$ (F)

The differential agretopicity (24) per gene is calculated as the log_2_ ratio of the tumor splicing antigenicity per gene divided by the normal splicing antigenicity per gene.

### The SpliceMutr pipeline—splice junction parsing

At its basic level, the SpliceMutr toolset takes as input a set of splice junctions in BED file format, their chromosome location, and splice-site coordinates. Per splice junction, SpliceMutr identifies whether the splice junction has a pair of splice sites that are found spliced together in the associated reference transcriptome. If so, the splice junction is classified as an annotated splice junction and is classified as an unannotated splice junction if not. If the set of splice junctions in the BED file has been previously annotated, as is the case with LeafCutter analysis, SpliceMutr will carry those specific annotations through to further analysis. LeafCutter-specific annotations of splice sites are detailed in Supplementary Fig. S1. Given supplied gene annotations, LeafCutter will identify specific splice junctions with respect to the reference. LeafCutter defines an annotated splice junction as a splice junction with both 5′ and 3′ splice sites matching known reference splice sites that are annotated as being spliced together. A cryptic splice junction is defined as having a 3′ splice site but not a 5′ splice site, a 5′ splice site but not a 3′ splice site, or both 3′ and 5′ splice sites that do not match known reference splice sites. A novel annotated splice junction is defined as a splice junction with 5′ and 3′ splice sites that match known reference splice sites that are not annotated as being spliced together.

### The SpliceMutr pipeline—transcript identification and splice-site modification

After splice junction parsing, the next step in the SpliceMutr pipeline is to identify the pair of flanking exons associated with each splice site for the target splice junction. Using each individual flanking exon, the upstream flanking transcripts are selected if they contain the upstream flanking exon, and the downstream flanking transcripts are selected if they contain the downstream flanking exons. All combinations of a single upstream transcript and a single downstream transcript are formed. In the case of a splice junction that is classified as annotated, only flanking transcripts with matching annotation names are preserved within the transcript combinations. Additionally, because the splice junction is annotated, the flanking exons should be directly spliced together within the transcript. If this is not the case, the matching flanking transcript pair is not preserved. With the unannotated splice junctions, all transcript pairings are preserved unless it is determined that there is a subset of matching transcript pairings that contain directly adjacent flanking exons. In this case, only those matching transcript pairings with directly adjacent flanking exons are preserved for analysis.

Once a set of candidate flanking transcript pairings are identified, transcript modification begins. To perform transcript modification, the full spliced upstream flanking transcript is joined to the full spliced downstream flanking transcript using the splice-site coordinates of the target splice junction as the joining point. If both the upstream and downstream flanking transcript have annotated untranslated regions (UTR) based on the reference transcriptome, then the joined transcript product is annotated as protein coding. This creates a joined transcript containing both five-prime and three-prime UTRs. If both the upstream and downstream flanking transcripts do not have annotated UTRs regions, then the joined transcript product is annotated as nonprotein coding. Now SpliceMutr performs ORF finding and translation of each joined transcript identified as protein-coding.

### The SpliceMutr pipeline—ORF finding and coding potential calculation

To perform ORF finding, SpliceMutr searches for the first start codon beginning at the five-prime UTR of the joined transcript. Once the first start codon is identified, SpliceMutr searches the transcript until the first stop codon is identified. The region from the first identified start codon to the first identified stop codon is extracted as the ORF for the transcript. Once the ORF is identified, the transcript is translated and saved for downstream analysis. ORF finding is performed this way to ensure that the experimentally validated ORF is conserved during transcript modification. All modifications to the ORF per target splice junction and joined transcript are recorded in the SpliceMutr metadata (Supplementary Fig. S1).

In the final stage of transcript modification, the coding potential of each target splice junction–modified transcript of size greater than or equal to 100 nucleotides is calculated using a modified version of the ORF Length and GC content (LGC) method (25). Only those genes with an average coding potential greater than zero are used in subsequent analysis.

### The SpliceMutr pipeline—MHC-binding affinity predictions and alternative and reference binder filtering

The set of proteins output from transcript modification is then processed, and MHC-binding affinity predictions are performed on tumor-specific and normal-specific kmers. The translated proteins are kmerized, and SpliceMutr then extracts out the unique set of kmers found in the set of translated proteins. SpliceMutr then uses MHCnuggets (version 2.3.2; ref. 22) to predict the raw binding affinity of each kmer using the HLA alleles specific to the samples being analyzed. SpliceMutr then extracts out those kmers with a predicted IC_50_ less than or equal to 500 nmol/L per HLA allele. Briefly, IC_50_ predictions are approximations of *in vitro* measurements of the concentration of a target peptide necessary to inhibit the binding of a high-affinity radiolabeled peptide to a specific MHC molecule by 50% (26). The lower this IC_50_ concentration, the higher affinity the target peptide has for the specific MHC molecule. If differential splice junction usage is calculated, predicted binders produced by tumor-specific transcripts are further filtered such that all binders also found within alternative normal-specific transcripts are removed. Predicted binders produced by normal-specific transcripts are filtered similarly to how tumor-specific binders are filtered, except all binders from the tumor-specific alternative event are filtered out. This filtering is not carried out when performing outlier splicing analysis. Additionally, all kmers found within the reference peptidome, as determined by a kmerized reference protein fasta, are filtered out. The protein fasta used was from the human GENCODE version 45 protein fasta file gencode.v45.pc_translations.fa.

### Gene and sample summaries of splicing antigenicity from RNA-seq analysis with SpliceMutr

In summary, SpliceMutr inputs sample HLA type, differentially used or outlier splice junctions, and variance-stabilized splice junction counts to calculate the number of filtered immunogenic kmers per splice junction–modified transcript and per sample. SpliceMutr then uses this information to calculate the per-gene splicing antigenicity metric. If differential splice junction usage is carried out, the differential agretopicity can be calculated per gene and used to filter for those genes with higher splicing antigenicity in tumor transcripts than normal. The per-gene splicing antigenicity metric can be calculated, which can be further summarized per sample by taking the average across all differential or outlier splicing-impacted genes. This provides a summary of splicing antigenicity per gene and further per sample for further statistical analysis.

### TCGA pan-cancer analysis

TCGA cancer subtypes were selected for analysis based on whether the cohort had at least six tumor and six normal samples with RNA-seq data. A total of 15 tumor subtypes fit this evaluation criterion: prostate adenocarcinoma, thyroid carcinoma, kidney renal clear cell carcinoma), lung squamous cell carcinoma, lung adenocarcinoma, breast-invasive carcinoma (BRCA), liver hepatocellular carcinoma, bladder urothelial carcinoma, kidney renal papillary cell carcinoma, colon adenocarcinoma, head and neck squamous cell carcinoma (HNSC), uterine corpus endometrial carcinoma, rectum adenocarcinoma, cholangiocarcinoma, and kidney chromophobe. Splice junction genomic coordinates and counts per tumor and normal sample in each cohort were extracted from recount3 (version 1.6.0; ref. 27) for LeafCutter (version 0.2.9; ref. 17) analysis. Those differential splice junctions were then run through SpliceMutr to generate a set of genes and their tumor-specific splicing antigenicity scores. Optitype HLA calls were used to obtain HLA genotypes per TCGA sample (28, 29) for SpliceMutr analysis.

We used the multiomic data from TCGA to further associate splicing antigenicity with common immunotherapy biomarkers. We specifically correlated the splicing antigenicity to a series of immune metrics, mutation-based metrics, and somatic mutation calls for each TCGA sample (28). The metrics used for correlative analysis were the T-cell receptor clonality, leukocyte fraction, number of immunogenic mutations, CIBERSORT (30) cell-type estimates, and the TMB calculated as the number of nonsilent mutations per megabase. The somatic mutation calls were used to determine mutations in SF3B1 per TCGA cancer subtype and sample (31). Using these data, we stratified TCGA samples per cancer subtype based on the existence of any nonsilent mutation first in SF3B1, then in 118 other splicing factor genes (31), and calculated whether the splicing antigenicity differed significantly between samples with mutations and samples without mutations using the Wilcoxon statistic and significance determined using Benjamini–Hochberg (BH)–adjusted *P* values below 0.05. Additionally, we stratified samples by mutation status per splicing factor gene and used linear regression to determine whether there were significant differences in splicing antigenicity between mutated and nonmutated samples for all TCGA samples while accounting for TCGA cancer subtype.

### Evaluating tumor-specific splicing antigenicity in a melanoma cohort treated with nivolumab, ipilimumab, or their combination

We obtained RNA-seq data from the previously described CM-038 melanoma immune checkpoint trial (32–34). This cohort contained biospecimens pre- and post-immunotherapy treatment in three trial arms. Patients in the first trial arm were given nivolumab and ipilimumab in combination (NIV1 + IPI3, *n* = 8). Patients in the NIV3-PROG arm (*n* = 32) had previous exposure to ipilimumab treatment and were only treated with nivolumab. Finally, patients in the NIV3-NAIVE arm (*n* = 27) had no previous anti-CTLA4 therapy and were only given nivolumab therapy. Patient response to treatment was evaluated using the RECIST 1.1 criteria (35). RNA-seq data for this cohort were preprocessed by and run through SpliceMutr to generate splicing antigenicity scores per gene impacted by outlier splicing. Whereas our previous studies performed differential splicing between tumor and normal samples, no normal samples were available for this cohort. Therefore, analysis of splicing differences was carried out within treatment arms with respect to RECIST 1.1 response type and treatment time using LeafCutterMD. Responder (CRPR), stable disease (SD), and progressive disease (PD) patient splice junction data were compared with baseline splice junction data using LeafCutterMD (Supplementary Figs. S2 and S3). The outlier splicing model in LeafCutterMD (19) was carried out to allow for uniform splicing analysis due to the small number of samples per response type in this cohort. LeafCutterMD output was then run through SpliceMutr to generate splicing antigenicity scores per gene and sample using the per-sample genotype generated from arcasHLA. The union of all splice junctions identified as being outlier splice junction per sample was combined to create the set of splice junctions for splicing antigenicity calculations. Once calculated, the splicing antigenicity was normalized by pseudo purity, but only those samples with pseudo purity greater than 0.01 were used for analysis. This resulted in dropping the lowest purity sample from analysis. When comparing the splicing antigenicity within treatment arm and across response type, averaged across genes or samples, the Wilcoxon test statistic with BH–adjusted *P* values (significance ≤0.05) was used. The nonadjusted Wilcoxon test statistic was used when comparing the splicing antigenicity across response type disregarding treatment arm. The paired Wilcoxon test was not used for this analysis because it would require missing gene splicing antigenicity values across conditions to be filled in with zeroes. The Cohens *d* is calculated similar to the effect size, except it uses a pooled standard deviation (36).

### Evaluating IEAtlas-validated peptides per BRCA sample

The Epitopes_In_Cancer_Tissues.txt file was downloaded from the IEAtlas (37) website, and the entirety of the peptides within the file was used as the validation peptide set. Per BRCA sample, the MHC-binding kmers determined using MHCnuggets were extracted and compared with the IEAtlas validation peptide set. The percentage validated per sample was then calculated.

### Statistical analysis

All statistical analyses in this article are performed in R (38). When testing for significant variation in the splicing antigenicity across conditions, the Wilcoxon test statistic with *P* values (significance ≤0.05) was used. Multiple testing (BH) correction was performed when greater than three comparisons are made. When testing the correlation between the splicing antigenicity and other metrics of response to immunotherapy, the Kendall Tau test statistic (significance ≤ 0.05) was used.

### Ethics approval and consent to participate

All TCGA data were used according to the TCGA guidelines on ethical usage. All sequencing data for the melanoma cohort followed ethical guidelines as previously published (32–34).

### Consent for publication

All patient sequencing data used in this analysis have been previously published and uploaded to Gene Expression Omnibus, Sequence Read Archive, and BioProject.

### Data availability

All TCGA data used for analysis in this article were recount3 (27) splice junction counts and metadata and The Immune Landscape of Cancer (28) open and protected Supplementary Material.

RNA-seq data for the melanoma cohort can be found at Gene Expression Omnibus: GSE91061; Sequence Read Archive: SRP094781; and BioProject: PRJNA356761.

The SpliceMutr pipeline is available from https://github.com/FertigLab/splicemutr or via zenodo (doi: 10.5281/zenodo.11116273).

**Project name:** SpliceMutr

**Project home page:** https://github.com/FertigLab/splicemutr

**Operating systems:** All analysis was performed on the CentOS Linux 7 (Core) or macOS 13.2.1

**Programming languages:** R, python, bash

**Other requirements:** R version 4.0.2 or higher, python 3.6.10 or higher

**License:** GNU General Public License v3

**Any restrictions to use by non-academics:** license needed

## Results

### Overview of the SpliceMutr pipeline for differential splicing antigenicity analysis

**Figure 1:**
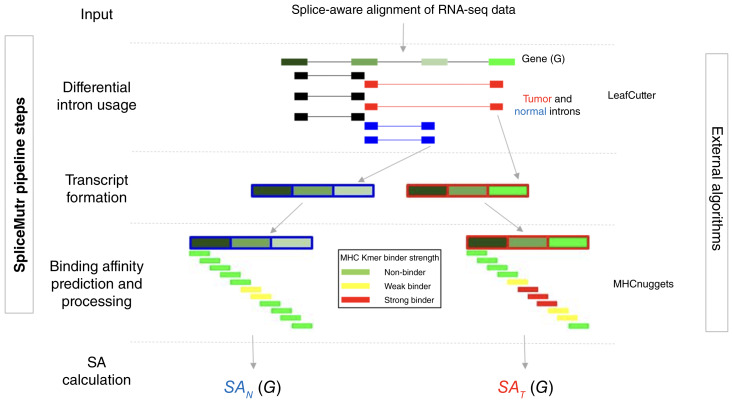
SpliceMutr pipeline. The SpliceMutr pipeline uses RNA-seq data from two groups of samples and evaluates the changes in SA between them. In this example, the alternative splicing analysis compares tumor and normal RNA-seq samples. The pipeline performs splicing-aware alignment, gene expression quantification, and HLA genotyping. The splicing-aware alignment splice junction counts are then input into LeafCutter to evaluate differential splice junction usage. The tumor-specific and normal-specific splice junctions undergo transcript formation, translation, and kmerization for MHCnuggets input through SpliceMutr, then are evaluated for genotype-specific MHC binders using MHCnuggets (22). MHC binders associated with normal-specific and tumor-specific peptides are then used to calculate a per-gene and sample SA metric dependent on the type of the sample. ${\;SA}_{T}\left(G\right)$ is calculated for tumor samples and ${SA}_{N}\left(G\right)$ is calculated for normal samples. SA, splicing antigenicity.

SpliceMutr is a comprehensive computational pipeline that identifies splicing-derived neoantigens through differential analysis of short-read RNA-seq data between two given groups of samples, illustrated for tumor and normal samples in Fig. 1. Due to replicates being required for the differential splicing analysis performed using the SpliceMutr pipeline, the tumor and normal sample inputs are unmatched in this study and should be for future applications. This pipeline leverages both R and Python scripts available from zenodo (doi: 10.5281/zenodo.11116273) to enable a new analysis approach for the identification of candidate neoepitopes. SpliceMutr relies on aligned RNA-seq reads using a splicing-aware aligner, such as STAR, and the class 1 HLA genotype per sample, which can be computed from the RNA-seq data (21). The split-read genomic coordinates and counts are then processed and used to perform differential or outlier splice junction usage analysis using LeafCutter (17). SpliceMutr uses the results of this differential splice junction usage analysis as the basis of transcript formation, ORF Length and GC content (LGC) coding potential prediction (25) translation, and peptide kmerization. Additionally, SpliceMutr uses the LeafCutter-identified differential splice junction clusters and their associated SpliceMutr-formed peptides to filter out kmer sequences shared between two groups of interest. Using the per-sample HLA genotype and the kmers associated with each transcript, SpliceMutr runs MHCnuggets (22) to predict MHC-binding affinity. Our choice of LeafCutter (17)and MHCNuggets (22) was based on LeafCutter’s benchmarking for differential splicing and MHCNuggets’ benchmarking involving MHC-binding prediction pan-cancer, respectively. Still, we note that the pipeline can be extended to other algorithms for differential splicing and antigen prediction. Nonetheless, the use of these robust tools enables us to apply the metrics resulting from the complete SpliceMutr pipeline to evaluate the downstream impacts of splicing on the immune landscape of tumors in large-scale cohorts of short-read RNA-seq data in this study.

### SpliceMutr infers mass spectrometry–validated peptide kmers after mitigating false positives through reference filtering

**Figure 2:**
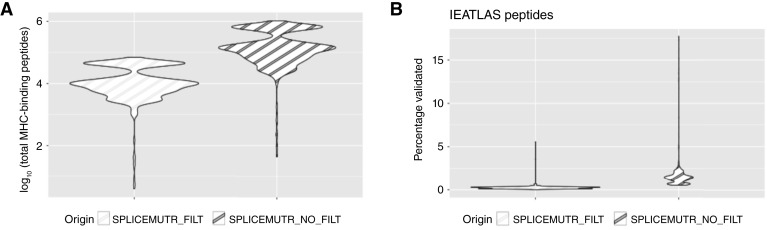
Comparison of predicted splicing antigens from SpliceMutr to proteomics datasets. **A,** The log_10_-transformed total number of MHC-binding kmers found with and without reference kmer filtering in all samples of the breast cancer TCGA cohort (BRCA). **B,** The percentage of IEAtlas-validated immunogenic kmers between SpliceMutr with and without reference kmer filtering in all samples of the breast cancer TCGA cohort (BRCA).

The cohort-level statistics for each sample’s splicing antigenicity estimated through SpliceMutr enables the comprehensive evaluation of its impact on the tumor immune landscape through analysis in TCGA. This analysis enables the quantification of antigenicity of all splice variants identified at a transcript level relative to normal samples in the atlas. Prior to leveraging our software for associations with tumor characteristics, we sought to evaluate its performance by determining whether the candidate splicing antigens are also observed in proteomics datasets. To minimize the number of false positive–binding kmers, we added an additional filtering step to remove all immunogenic kmers that were also found within the human GENCODE reference proteome (version 45), ensuring that only kmers derived from non-annotated splice junctions were used in the calculation. When comparing SpliceMutr with reference filtering and SpliceMutr without reference filtering, we see that SpliceMutr without reference filtering has more total MHC-binding peptides [mean = 233,202 (0–1,038,488)] identified than does SpliceMutr with reference filtering [mean = 16,381 (0–69,691; Fig. 2A)]. We then sought to determine if any candidate splice antigens are binding to MHC molecules. To perform this analysis, we leveraged IEAtlas (37). IEAtlas is a repository containing predicted MHC-binding peptide antigens derived from noncoding ORFs that are validated as MHC-eluted peptides. In the analysis shown in Fig. 2B, we use the IEAtlas repository of validated peptides as a set of peptides to use for validation of neoantigens learned from our analysis of short-read RNA-seq data [mean of filtered = 0% (0%–5.56%), mean of non-filtered = 1.56% (0.53%–17.72%)]. For this analysis, we compare the results from SpliceMutr in the breast cancer (BRCA, 1,094 samples) TCGA cohort per sample, in which the number of validated IEAtlas kmers found to be MHC-binding kmers is determined by the HLA genotype per sample. We chose the BRCA TCGA cohort for validation analysis because it was one of the largest TCGA cohorts available. We observe that some of our candidate splice antigens validate in IEAtlas; however, the proportion of validated antigens is less than 1% with or without filtering. We attribute the relatively low validation rate to inconsistency between RNA and protein, and incomplete annotation in the IEAtlas database. Given the relatively similar proportion of validation despite the decreased number of splicing antigens, we hypothesize that the reference protein filtering reduces the number of false positives justifying our use of this filtering in the remainder of this study.

To benchmark the relative rate of validation for use of this algorithm, we sought to compare our results to those obtained from NeoSplice (14). Whereas SpliceMutr is a cohort-level analysis technique, NeoSplice estimates antigenicity of splice variants in a single tumor sample relative to its reference normal. Therefore, we sought to compare performance in the case of the tumor with a large number of MHC-binding kmers from SpliceMutr (BRCA with 49,356 kmers). Whereas SpliceMutr identified 68 antigens in IEAtlas, NeoSplice inferred only 1. Although this difference may arise due to larger total number of splice variants detected in SpliceMutr than in NeoSplice, we note that both point to the need for enhanced ground truth databases for training and testing antigens arising from transcript variants as a novel source of candidate antigens.

### The splicing antigenicity metric extends SpliceMutr from cohort-level splicing antigens to patient-level statistics of the splicing antigen burden

As we describe above, SpliceMutr uniquely estimates splicing antigens on a cohort level. However, to evaluate the impact of these antigens on the tumor microenvironment or in relation to immunotherapy biomarkers still requires a patient-level statistic. After predicting antigenicity of each transcript, SpliceMutr outputs splicing antigenicity metrics per gene and per sample to identify neoantigen candidates and compare splicing antigenicity with respect to other categorical or covariate data related to the genes or samples of interest. The splicing antigenicity is a measure of the potential for tumor-specific or normal-specific splicing variants to create antigens, and the tumor splicing antigenicity is normalized by the tumor purity. The higher the tumor splicing antigenicity of a gene of interest, the more likely the gene of interest harbors splicing variants with the potential for being useful antigens. We note that these metrics and, thus, downstream analysis depend on the specific algorithms selected for analysis in the pipeline.

The gene splicing antigenicity metric is calculated as follows:

$${SA}_{T}\left(G\right)\;=\;\frac{\sum_{\forall i\;\in I}\;\left(R_{i}\cdot \frac{k_{Ti}}{k_{i}}\right)}{\left|I\right|},$$ (G)

$${SA}_{T,norm}\left(G\right)\;=\;\frac{\sum_{\forall i\;\in I}\;\left(R_{i}\cdot \frac{k_{Ti}}{k_{i}}\right)}{P*\left|I\right|},$$ (H)

$$SA_{N}\left(G\right)=\frac{\sum_{\forall i\;\in I}\;\left(R_{i}\cdot \frac{k_{Ni}}{k_{i}}\right)}{\left|I\right|},$$ (I)

$$SA_{N,norm}\left(G\right)=\frac{\sum_{\forall i\;\in I}\;\left(R_{i}\cdot \frac{k_{Ni}}{k_{i}}\right)}{P*\left|I\right|},$$ (J)

in which ${SA}_{T}\left(G\right)$ is the tumor splicing antigenicity for gene *G*, $R_{i}$ is the variance-stabilized splice junction read count, $k_{Ti}$ is the number of tumor-specific binders from the peptide associated with the transcript modified by splice junction i, and I is the number of tumor-specific splice junctions for gene G. Using parameters obtained from normal samples and normal sample splicing patterns, a normal splicing antigenicity, ${SA}_{N}\left(G\right)$ can be calculated. In ${SA}_{N}\left(G\right)$, $R_{i}$ is the variance-stabilized splice junction read count, $k_{Ni}$ is the number of normal-specific binders from the peptide associated with the transcript modified by splice junction I, and I is the number of normal-specific splice junctions for gene G. ${SA}_{T,norm}\left(G\right)$ is the tumor purity normalized tumor splicing antigenicity for gene *G* in which *P* is the tumor purity. The splicing antigenicity can then be used to calculate a differential splicing antigenicity per gene between normal and tumor splicing patterns using a log ratio, it can be used to create a splicing antigenicity gene rank per sample, and it can be averaged to create a splicing antigenicity score per sample across all genes. From here on out, splicing antigenicity will refer to Eq. (H) and Eq. (H) is used when calculating the splicing antigenicity within TCGA and within the ICI-treated melanoma cohort.

### Splicing antigenicity displays widespread pan-cancer variability and is anticorrelated with TMB

**Figure 3:**
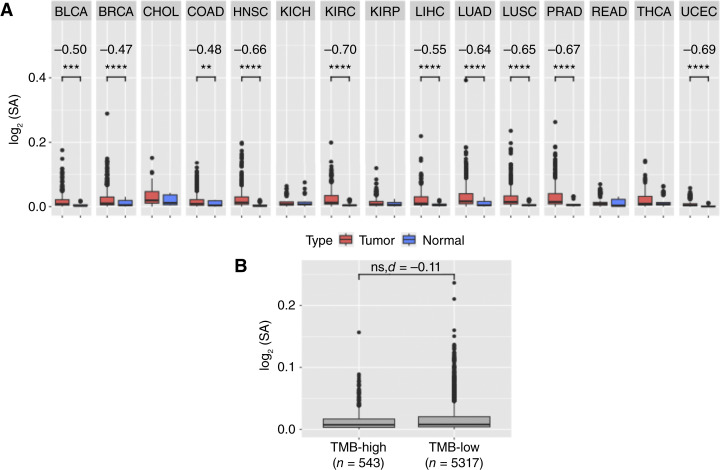
SA by tissue type and in relation to the tumor mutational burden. **A,** The SA averaged across genes per sample for tumor and normal samples for each tumor type analyzed (Wilcoxon test, Cohens *d*). **B,** The SA averaged across all genes per sample for TMB=high and TMB-low samples for all analyzed TCGA samples (linear regression, Cohens *d*). **, *P* value ≤ 0.005; ***, *P* value ≤ 0.0005; ****, *P* value ≤ 0.00005. See TCGA cancer-type abbreviations in Supplementary Table S1. SA, splicing antigenicity.

To evaluate the prevalence of splicing antigenicity in a pan-cancer manner, we first evaluated the tumor versus the normal splicing antigenicity in each sample in TCGA (Fig. 3A). Among the tumor types analyzed, we observed a trend toward increased splicing antigenicity in tumor versus normal samples pan-cancer, which is statistically significant (measured by Wilcoxon) and higher in tumor samples for bladder urothelial carcinoma (*P* value = 2.80E−04, Cohens *d* = 0.50), BRCA (*P* value = 2.10E−10, Cohens *d* = 0.47), colon adenocarcinoma (*P* value = 1.78E−03, Cohens *d* = 0.48), HNSC (*P* value = 1.70E−13, Cohens *d* = 0.66), kidney renal clear cell carcinoma (*P* value = 4.90E−14, Cohens *d* = 0.70), liver hepatocellular carcinoma (*P* value = 9.60E−07, Cohens *d* = 0.55), lung adenocarcinoma (*P* value = 2.40E−13, Cohens *d* = 0.64), lung squamous cell carcinoma (*P* value = 8.10E−12, Cohens *d* = 0.65), prostate adenocarcinoma (*P* value = 3.80E−15, Cohens *d* = 0.67), and uterine corpus endometrial carcinoma (*P* value = 1.50E−08, Cohens *d* = 0.69). All tumor subtypes with significant differences in splicing antigenicity across tumor and normal samples have a higher splicing antigenicity in the tumor samples.

The observation of increased tumor splicing antigenicity in many cancer subtypes leads us to hypothesize that the total level of splicing antigens in a tumor may impact its immunogenicity and immunotherapy response. To evaluate the connection of splicing antigenicity to established biomarkers for immunotherapy pan-cancer, we next stratified all TCGA samples in TMB-high (TMB ≥10 mutations/Mb) and TMB-low (TMB <10 mutations/Mb) subgroups (4) and quantified differences in their splicing antigenicity averaged across genes per sample (Fig. 3B). We find that TMB-high tumors have insignificantly lower splicing antigenicity than TMB-low tumors (*P* value = 0.241, Cohens *d* = −0.11, Wilcoxon test). We also correlated the splicing antigenicity to CIBERSORT immune cell deconvolutions (Supplementary Fig. S5A) to assess whether the splicing antigenicity was associated with any immune cell compositions and find that there are no associations between the splicing antigenicity and immune cell composition. Altogether, these findings support the conclusion that tumor-associated splicing variants have more immunogenic potential than normal, and that the splicing antigenicity is insignificantly correlated with the TMB based on Fig. 3B, and that the splicing antigenicity does not correlate with any specific immune cell component in TCGA tumors. We hypothesized that the splicing antigenicity would be uncorrelated with the TMB except for high TMB tumors where the splicing machinery may be disrupted due to mutations. To further test this hypothesis, we explored the association of splicing antigenicity per tumor type across all samples to TMB (Supplementary Figs. S4 and S5A). In this comparison, we found that the median splicing antigenicity per tumor type in TCGA is insignificantly negatively correlated to the median TMB per tumor type in TCGA (*P* value = 0.63, tau = −0.1; Kendall Tau Test). We also do not observe any significant correlations between splicing antigenicity and TMB for any tumor subtype in the TCGA cohorts analyzed (Supplementary Fig. S5B). These results warrant further analysis within tumor subtypes to determine the impact of tumor splice variants on immunogenicity and to test whether splicing-derived antigens act to supplement mutation-derived antigens in TMB-low tumors.

### The abundance of splicing antigens associates with the frequency of mutations in the splicing machinery

**Figure 4:**
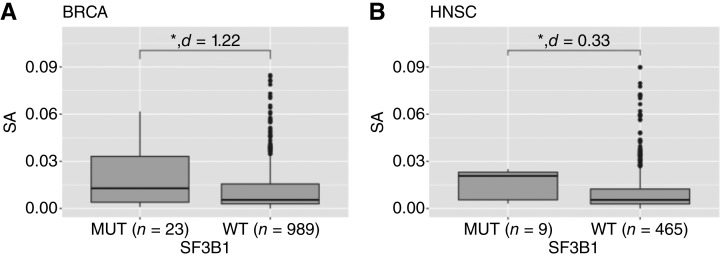
Pan-cancer correlation analysis of per-sample SA with nonsilent mutations in splicing machinery coding genes. Distribution of SA per sample, averaged across genes, by mutations in the splicing factor gene SF3B1 in BRCA (**A**) or HNSC (**B**). *, *P* value ≤ 0.05. See TCGA cancer-type abbreviations in Supplementary Table S1. SA, splicing antigenicity.

Mutations in the catalytic RNA-core (6, 39) and in scaffold splicing factor proteins (9, 40–42) have been associated with splicing alterations. To examine potential links between mutations in the splicing machinery with splicing antigenicity across tumor types, we first categorized samples in TCGA based on the presence of nonsilent mutations in the splicing factor genes SF3B1 (43) and analyzed levels of splicing antigenicity (Supplementary Table S2). The only cancer subtypes that showed significant differences in splicing antigenicity were between SF3B1-wt and mutant samples in BRCA and HNSC (Fig. 4A and B; Supplementary Table S2), with higher splicing antigenicity in mutant samples (BRCA *P* value = 0.017 and Cohens *d* = 1.22; HNSC *P* value = 0.05 and Cohens *d* = 0.33, Wilcoxon test). These samples did not show BH–adjusted significant differences in splicing antigenicity when adjusted across all comparisons for the 119 cancer associated splicing factor genes (31) per TCGA cancer subtype. This observation is consistent with the hypothesized role of SF3B1 as a biomarker for immunotherapy currently under evaluation in a breast cancer clinical trial (44) but suggests that it may not generalize to additional cancer subtypes except for head and neck cancer.

### Response to immunotherapy in patients with melanoma is associated with increased splicing antigenicity

Although the TCGA analysis enables us to correlate splicing antigenicity to various biomarkers of ICI response pan-cancer, including TMB, this database is only composed of pretreatment samples and does not allow the direct comparison of splicing antigenicity before and after treatment. Therefore, to directly associate splicing immunogenicity with ICI response, we applied SpliceMutr to a cohort of RNA-seq data from 67 patients with metastatic or advanced melanoma treated as part of an ICI clinical trial described previously (32–34). Briefly, this cohort has RNA-seq data pre- and post-treatment in patients from three arms. Patients in the NIV1 + IPI3 arm were treated with nivolumab (anti–PD-1) and ipilimumab (anti-CTLA4) in combination (*n* = 8 patients) and had no previous exposure to ipilimumab. In the NIV3-PROG arm, patients were previously exposed to ipilimumab and were only treated with nivolumab after progression while on ipilimumab (*n* = 32 patients). In the NIV3-NAIVE arm, patients had no previous anti-CTLA4 therapy and were only given nivolumab as a monotherapy (*n* = 27 patients).

This melanoma cohort contains only tumor samples, but not their noncancer controls, limiting an analogous comparison of differential splicing used in the TCGA. To account for the smaller sample size and a lack of normal controls in this melanoma cohort, we used LeafCutterMD (19) to perform a one versus ipilimumab-naïve baseline outlier analysis (Supplementary Figs. S2 and S3). The outlier analysis method using LeafCutter makes comparing splicing differences between conditions with small sample sizes, in contrast to the requirement of six replicates per condition in the differential splicing analyses used for TCGA. This outlier splicing analysis is useful for early-phase clinical trial analysis where there are typically a small number of responders for comparison against nonresponders. Using LeafCutterMD, we identified 6,674 total genes impacted by outlier splicing. Using this subset of genes, we calculated the mean splicing antigenicity per sample, and we used the Wilcoxon test *P* values to determine significant differences in splicing antigenicity within treatment arm and across objective response (Fig. 4). The CRPR group includes all complete and partial responders, the SD group includes all SD patients, and the PD group includes all PD patients.

**Figure 5:**
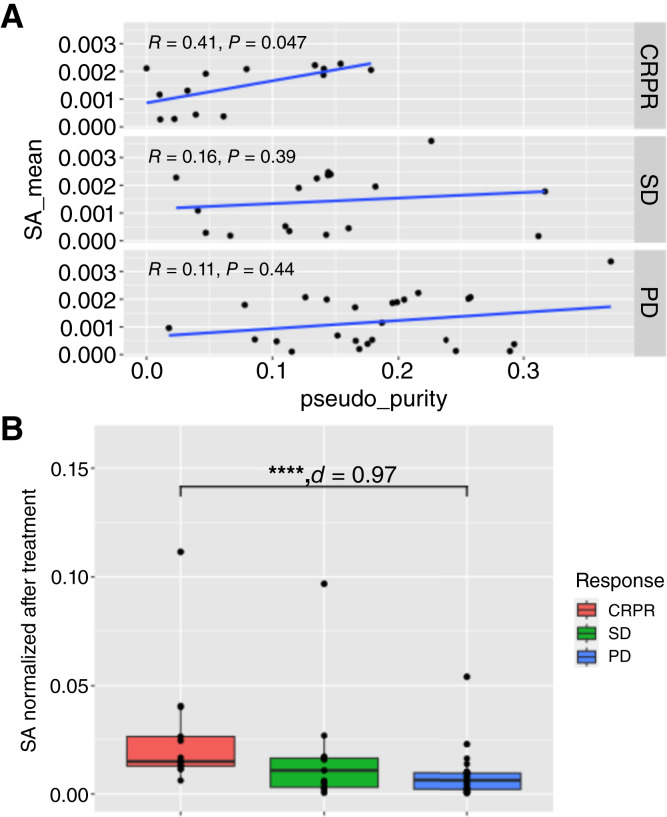
Per-patient SA of ICI-treated patients with melanoma per treatment arm and response type after treatment. **A,** The per-patient pseudo purity compared with the mean SA averaged across genes by the response for all treatment arms. Kendall Tau test. **B,** The mean SA averaged across genes per patient and normalized by the pseudo purity, for all treatment arms combined. Wilcoxon test and Cohens *d*. ****, *P* value ≤ 0.00005. SA, splicing antigenicity.

Whereas TCGA tumors were obtained from surgical biospecimens and quality controlled to ensure tumor purity prior to sequencing, this clinical trial uses biopsies for sequencing. The resulting variability in tumor purity in these biospecimens may impact the splice variants detected and subsequent estimates of splicing antigenicity. To evaluate the impact of the therapeutic effect on the splicing antigenicity metric, we plotted the ESTIMATE-calculated pseudo purity (23) versus the splicing antigenicity (Fig. 5A). We found that the pseudo purity was significantly positively correlated with the splicing antigenicity in responders (Kendal Tau test, *P* value = 0.047, tau = 0.41). Additionally, we found that the splicing antigenicity was significantly correlated with the ESTIMATE-calculated immune score for PD patients (Supplementary Fig. S6). This ESTIMATE-calculated pseudo purity observation motivated our normalization of the splicing antigenicity, per sample, by the pseudo purity by modifying Eqs. (G) and (H) on all the results presented in this study, including the TCGA analyses described in the previous section. Once normalized, we evaluated the splicing antigenicity independent of treatment arm across response (Fig. 5B). We found that responders had a significantly higher splicing antigenicity than PD patients (Wilcoxon test, *P* value = 7.06E−05, Cohens *d* = 0.97; Fig. 5B). Although we do not see the same trends prior to treatment (Supplementary Fig. S2), significance in responders, at least for the NIV-PROG arm, is not reached due to outlier samples and a minimal number of responders available for comparison. These results show that responders have a higher immunogenic potential.

**Figure 6:**
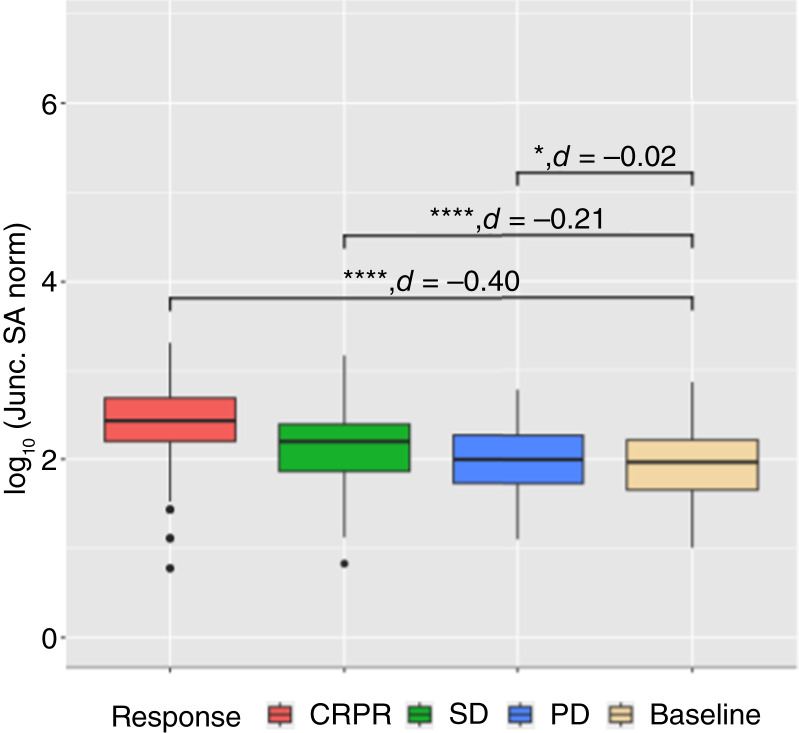
Per splice junction normalized SA for responders compared with baseline samples. The SA averaged across samples for the subset of splice junctions derived from the top 20 genes with the highest SA per sample. The black horizontal line is the median SA for baseline samples. *, *P* value ≤ 0.05; **, *P* value ≤ 0.005; ***, *P* value ≤ 0.0005; ****, *P* value ≤ 0.00005. SA, splicing antigenicity.

To validate the pairwise relationship between the normalized splicing antigenicity and response to ICI therapy in this melanoma cohort, we extracted the splice junctions associated with the union of the top 20 genes, by splicing antigenicity, impacted by outlier splicing per sample. Then, we averaged the splicing antigenicity per splice junction across samples by response to obtain a mean splicing antigenicity per splice junction. We then performed a pairwise Wilcoxon test between the splicing antigenicity of the same splice junctions in responders compared with baseline, SD patients compared with baseline, and PD patients compared with baseline. We found that responders (*P* value = 1.06E−33, Cohens *d* = −0.40; Fig. 6), SD patients (*P* value = 1.34E−32, Cohens *d* = −0.21; Fig. 6), and PD patients (*P* value = 1.61E−2, Cohens *d* = −0.02; Fig. 6) have a significantly increased splicing antigenicity compared with baseline with PD patients having a splicing antigenicity very similar to baseline. Furthermore, there is a decreasing splicing antigenicity as response worsens (Fig. 6). These results further suggest that the response in this cohort is in part mediated by the immunogenic potential of splicing antigens.

## Discussion

This pan-cancer evaluation of splicing antigenicity using SpliceMutr demonstrates that the splicing antigenicity is higher in tumor samples than that in normal samples and is weakly and insignificantly negatively correlated with TMB. Additionally, this study shows that in some tumor types, mutations in the splicing machinery significantly alter the tumor splicing antigenicity of samples that harbor these mutations. The evaluation of SpliceMutr splicing antigenicity in the melanoma cohort treated with ICI therapy shows that the tumor splicing antigenicity is increased in responders relative to nonresponders and that responders are characterized by increased splicing antigenicity per splice junction after ICI therapy relative to before.

Within our TCGA analysis, we were only able to analyze 15 TCGA cancer subtypes due to a lack of a minimum number of six normal samples in the other cancer subtypes. Had we used a pan-normal reference from TCGA or GTEX, we might have been able to analyze all tumor samples from TCGA. Yet, creating a pan-normal reference might introduce normal splice junctions into the analysis that are tissue-specific, eliminating the tumor specificity of the analysis. Moreover, there are confounded batch effects between normal samples from different tissue sites in the TCGA. This would dull the impact of localized tumor-specific splicing patterns. Furthermore, the normal splicing patterns do play a role in the tumor splicing antigenicity. The tumor antigenicity does include differential analysis from normal samples in the form of tumor-specific binding kmer filtering. Binding kmers are deemed tumor-specific if none of the binding kmers associated with normal splicing patterns are found in the set of binding kmers associated with tumor-specific splicing patterns. This is reversed for normal-specific binding kmers.

Our evaluation of splicing antigenicity using the RNA-seq data from immunotherapy-treated melanoma samples in the CM-038 clinical trial (32–34) demonstrates that splicing antigenicity is significantly increased in responders relative to nonresponders. Furthermore, our work shows that those patients that respond well to treatment have a larger difference between the splicing antigenicity after treatment relative to baseline compared with those that respond poorly. These findings are contradictory to the findings in (15), where their calculation of the number of splicing-derived tumor neoantigens showed no significant variation in the response in a cohort of melanoma patients treated with ICI therapy. We attribute this discrepancy to the fact that although SpliceMutr uses the number of splicing-derived neoantigens as a basis for the splicing antigenicity score, SpliceMutr generates a sample-level splicing antigenicity score dependent on several additional and relevant aspects of the splicing neoantigens other than just the number such as the sample tumor purity, the variance-stabilized intron read count, and the total number of kmers in the intron-modified transcript*.* Our work is also contradictory to the findings by (5) where they show that the tumor variant burden, a metric that includes sources of tumor-specific RNA variants in the calculation of variant burden, does not positively contribute to predicting response to ICI therapies over standard measures of TMB. The study by (5) differs from this work in their definition of what constitutes a tumor-specific splice junction though. The work by (5) filters out all splice junctions found within any normal TCGA or GTEX samples whereas we do not carry out this filtering step. The SpliceMutr reference filtering is carried out after prediction of MHC-binding kmers. Another difference that may account for the discrepancy between (5) and this work is the calculation of the splicing antigenicity. SpliceMutr aggregates several metrics into the splicing antigenicity score prior to the analysis shown within the ICI-treated melanoma cohort. Additionally, in the Lu and colleagues article (13), they find that modulation of splicing factor function in combination with ICI therapy results in the development of splicing-derived neoantigens that can cause an immune response. Our finding that mutations in the splicing factor machinery can result in increased splicing antigenicity in some cancers relative to wild-type samples is complemented by this study even though SF3B1 does not reach BH-adjusted *P* value significance in our study. As a result, our study shows that splicing antigenicity can be increased in some cancers even without drugged modulation of the splicing factor machinery. Altogether, these findings point to a model in which splicing contributes to the antigen landscape in tumors, thereby increasing the ability of immune recognition and immunotherapy response.

Supplementary Figure S8 details the splicing antigenicity per treatment arm after treatment and shows that the only arm to show stratification of splicing antigenicity by response is the ipilimumab progressed arm. The ipilimumab-naïve arm does not show stratification of the splicing antigenicity by response. The reports by (45, 46) both show that previous exposure to anti–CTLA-4 modifies the biomarkers associated with response to anti–PD-1 therapy and proves favorable with respect to response to anti–PD-1 therapies. The splicing antigenicity, similar to the biomarkers reported by (45, 46), only shows significant differences between PD patients and responders and SD patients and responders within the anti–CTLA-4 exposed patients after treatment. Therefore, we believe that the splicing antigenicity may prove to be a reliable predictor of response to ICI therapy in melanoma following further exploration. Yet, determination of the splicing antigenicity’s association with the metrics derived by (45, 46) is either impossible due to not having matched DNA sequencing information for the melanoma cohort or unreasonably underpowered due to the small sample size of the cohort we are analyzing. In the future, the development or curation of a larger cohort of ICI-treated patients with melanoma including DNA sequencing would aid in the evaluation of the splicing antigenicity.

Evaluating splicing neoantigens in cancer has been explored by several key studies (11, 13, 15, 43). Yet, each method focuses solely on the number of splicing-derived neoantigens produced and uses an outlier-based approach dependent on tissue-matched GTEx (47) samples as reference (11), an outlier-based approach dependent on the entire set of GTEx samples as normal (15), or an approach based on the differential isoform usage of reference isoforms (13). The analysis of an independent reference in these previous studies may introduce batch effects in the evaluation of splicing antigens. SpliceMutr addresses this issue, by using normal samples from the same tissue site within the differential TCGA analysis splicing analysis performed. Still, further work is needed to make larger cohorts of normal samples generated in the same technical batch and with true normal samples that may not be subject to the splicing alterations resulting from field effects in matched normal samples.

The SpliceMutr pipeline benefits from the introduction of several new elements to the analysis of splicing-derived neoantigens. SpliceMutr uses LeafCutter differential splice junction analysis as well as LeafCutterMD outlier splicing analysis to identify dysregulated splicing targets in cancer. By using these tools, SpliceMutr leverages the strengths of short-read RNA-seq: primarily high coverage, minimizes the pitfalls of short-read RNA-seq: non–multiexon-spanning reads, and allows for the response of samples across conditions. SpliceMutr also introduces calculations of nonsense-mediated decay and subsequent filtering of noncoding transcripts into the evaluation of the alternatively spliced or outlier spliced junctions. SpliceMutr enables calculation of a tumor purity–normalized splicing antigenicity score that enables evaluation of the impact of a tumor’s immunogenicity and can be used as an immunotherapy biomarker. Additionally, by using the variance-stabilized splice junction read count in the calculation of the splicing antigenicity per sample, SpliceMutr transitions its cohort-based differential splice junction usage analysis through LeafCutter into a sample-specific analysis. Those splice junctions identified at the cohort level as being differentially used between tumor and normal samples that have low expression in a sample will contribute less to the gene-level splicing antigenicity metric that is calculated per sample.

SpliceMutr is unique as a splicing antigen tool in computing cohort-level differentially used splice junctions for antigen prediction. In contrast, the alternative splicing-derived neoantigen prediction pipelines, such as NeoSplice (14) and ISOTOPE (15), provide single patient-level statistics. Moreover, NeoSplice requires a single tumor and matched normal sample limiting analysis as in our ICI-treated cohort. We found that a SpliceMutr uses LeafCutter to determine differentially used splice junctions and then calculates a sample, or HLA genotype, specific MHC-binding kmer profile, the MHC-binding kmers that can be used for comparison to the neoantigen output of NeoSplice represent a semi sample-specific representation of the cohort-level splicing signatures. However, NeoSplice outputs a sample-specific neoantigen profile. A way to perform this type of comparison would be to modify SpliceMutr to consider the variance-stabilized split-read count during the MHC-binding kmer-filtering step. This would ensure that per sample, only those MHC-binding kmers derived from split-reads with high expression would pass through the filtering step. We believe that including the variance-stabilized split-read count in the calculation of the splicing antigenicity provides for a more dynamic and sample-specific metric and is an important area of future extension to SpliceMutr beyond the scope of this current study.

Additionally, differentially used splice junctions are identified at the cohort level within SpliceMutr. Yet, SpliceMutr uses the variant-stabilized intron read count when calculating the splicing antigenicity per gene and per sample. This ensures that splice junctions with low expression factor in far less than do splice junctions with high expression for the sample. Using the variance-stabilized intron read count also ensures that the splicing antigenicity is a sample-level metric, despite being derived from cohort-level differentially used splice junctions. Despite this, the cohort-level evaluation of the differentially used splice junctions make comparison of SpliceMutr to other alternative splicing-derived neoantigen prediction pipelines, such as NeoSplice (14) or ISOTOPE (15) difficult. NeoSplice and ISOTOPE provide single patient-level statistics. NeoSplice is a pipeline that processes a single tumor and matched normal sample and predicts tumor-specific splicing-derived neoantigens and outputs a sample-specific neoantigen profile. However, SpliceMutr generates sample-specific representation of the cohort-level splicing signatures. Therefore, a direct comparison of the predicted neoantigens between SpliceMutr and NeoSplice is not appropriate. A way to perform this type of comparison would be to modify SpliceMutr to consider the variance-stabilized split-read count during the MHC-binding kmer-filtering step. This would ensure that per sample, only those MHC-binding kmers derived from split-reads with high expression would pass through the filtering step. We believe that including the variance-stabilized split-read count in the calculation of the splicing antigenicity provides for a more dynamic and sample-specific metric though.

In spite of these differences, both SpliceMutr and NeoSplice uncover a relatively low proportion of splicing antigens as validated MHC peptides in IEAtlas. This observation points to limitations in both antigen prediction algorithms from kmers associated with transcriptomic variants and reference databases for their validation that will provide a limitation for all splicing antigen algorithms beyond the scope of the current work to address. As improved antigen prediction methods for transcript variants develop, we hypothesize that these splicing antigenicity metrics will still be applicable to the resulting splice antigen prediction methods to enhance correlative and biomarker analyses of immunotherapy studies.

Although useful for evaluating the impact of splicing on the neoantigen burden, this study does have limitations. The SpliceMutr pipeline does not perform transcriptome assembly, instead transcripts are modified by an individual splice junction of interest. This does not allow for multiple alternative or noncanonical events to be included in a single transcript. Additionally, SpliceMutr does not include analysis of the intron retention alternative splicing event in differential analysis or transcript formation. This is due to the desire to perform differential and outlier splice junction usage analysis using LeafCutter and LeafCutterMD, each of which defines splice junctions as exon-spanning reads. Intron retention events, if able to escape NDM, can result in immunogenic peptides. The lack of intron retention detection results in an underestimation of the splicing antigenicity for some genes. Although it might be possible to identify retained introns using tools such as IRFinder (48) and iREAD (49), label retained introns using codes and then combine the identified intron retention splice junctions with exon-spanning splice junctions as input into LeafCutter and LeafCutterMD. This would result in differential or outlier splicing analysis that includes intron retention while using the LeafCutter or LeafCutterMD framework. Future work extending this approach to splicing inferred from long-read sequencing may overcome some of the limitations of this pipeline resulting from the reliance on inferred transcripts from short-read sequencing.

Although developing this pipeline for bulk RNA-seq data enables analysis of large-scale tumor cohorts, low tumor purity or mixtures of additional cell types can impact our estimates of splice variants. In this study, we address this by scaling our estimate of splicing antigenicity by tumor purity. Single-cell profiling can overcome this need for scaling by isolating tumor cells for transcriptional profiling but estimates of splice variants from this technology remain challenging due to high dropout of RNA signal. Therefore, future studies relying on profiling of malignant epithelial cells as through laser capture microdissection or improved high-coverage single-cell technologies are essential to overcome these limitations.

A further limitation of this study is that SpliceMutr relies on universal MHC-binding affinity thresholds to establish antigens and does not leverage immunogenicity calculation. Using MHC-specific IC_50_ thresholds when predicting peptide binders improves the accuracy of the prediction (50). Yet, due to using a uniform threshold for peptide binders, less than or equal to a 500 nmol/L IC_50_ score, SpliceMutr does not allow for custom MHC-specific binder classification. Custom MHC-specific binder classification can be implemented by including an internal lookup table to appropriate MHC-specific binder thresholds during the binding affinity prediction and selection phase. We decided not to use immunogenicity calculations within this study and instead focus on the MHC to peptide binding as the minimum requirement for splicing-derived antigens to be recognized by the immune system. We make this choice due to the abundance of MHC peptide–binding affinity measurements available as training data for the MHC=binding prediction algorithms relative to the limited available data for T-cell receptor MHC peptide–binding algorithms. Furthermore, a recent study by (51) established BigMHC, a tool that performs immunogenicity calculations. Extensions of SpliceMutr will take the output of this immunogenicity predictor as input into the binding peptide filtering stage of SpliceMutr. Yet, the tool that we decided to integrate into SpliceMutr currently, MHCnuggets, has equivalent or better performance than NetMHCpan when compared in 2019, has trained models for 299 MHC alleles, and uses a “super-allele” approach to make predictions on rare alleles, which are not included in the base set of models. When a rare allele is input to the software, it is matched with the model for the closest allele, based on allele clustering by binding pocket biochemical similarities, experimental mass spectrometry, and binding assay results. Still future work validating the relative performance of these methods in the context of splicing antigens is still needed, and we note that the splicing antigenicity metrics introduced in this study could readily be adapted to alternative antigen prediction methods.

SpliceMutr’s reliance upon LeafCutter differential splicing analysis means that there is a minimum number of normal samples required to carry out the differential analysis. This means only 15 TCGA cancer subtypes were analyzed in this study due to low numbers of normal samples in the other 18. To analyze all 33 TCGA cancer subtypes, we could have leveraged a pan-normal dataset during differential analysis. We decided against using a pan-normal dataset as this method might have introduced normal splice junctions into the analysis that are tumor-specific in another tissue type therefore dulling the impact of localized tumor-specific splicing patterns.

## Supplementary Material

Supplementary Table 1 Supplementary Table S1. TCGA cancer study abbreviations and names.

Supplementary Table 2 Supplementary Table S2. Splicing antigenicity differences between samples with mutated and samples without mutated splicing factor genes. The splicing antigenicity differences between samples with mutated and non-mutated splicing factors for all 119 chosen splicing factor genes and for each TCGA cancer subtype.

Supplementary Data The supplementary figures file
