# Supplementary material for: SpliceMutr Enables Pan-Cancer Analysis of Splicing-Derived Neoantigen Burden in Tumors
Source: Cancer Res Commun. 2024 Dec 16;4(12):3137–50. doi: 10.1158/2767-9764.CRC-23-0309 (PMC11648103; doi:10.1158/2767-9764.CRC-23-0309)
Supplement: Supplementary Data — The supplementary figures file [file crc-23-0309_supplementary_data_suppsd.docx]

**Supplement**


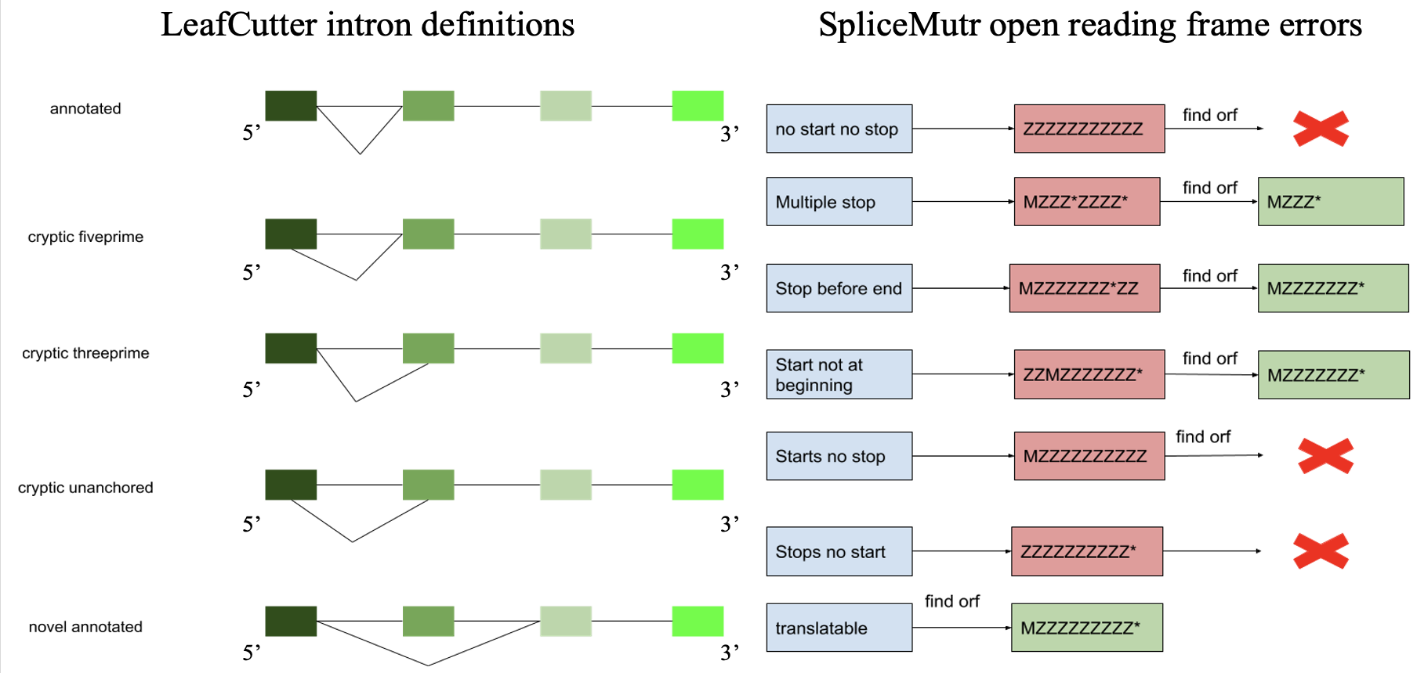


**Supplementary Figure S1. LeafCutter intron definitions and SpliceMutr open reading frame errors.** The LeafCutter intron definitions are a visual representation of the LeafCutter intron definitions with respect to a mock gene model. Annotated introns have a pair of splice sites located at the genomic coordinates of a documented intron. Cryptic intron types have either one or both splice sites located at genomic coordinates not associated with a documented intron. Novel annotated introns have a pair of splice sites individually located at the genomic coordinates of documented introns but not at splice site locations documented to be joined together. The splicemutr open reading frame errors are a visual representation of the open reading frame (ORF) errors that splicemutr documents during transcript formation. The full range of the DNA equivalent of stop codons are defined as such during transcript formation, but this example only features the TAG stop codon. If an open reading frame can be found after modification of a reference transcript by a differentially used intron, then the transcript is translated with the appropriate error documented. If the modified transcript has unchanged ORF start and end codons relative to the reference, then the modified transcript is documented as being immediately translatable.


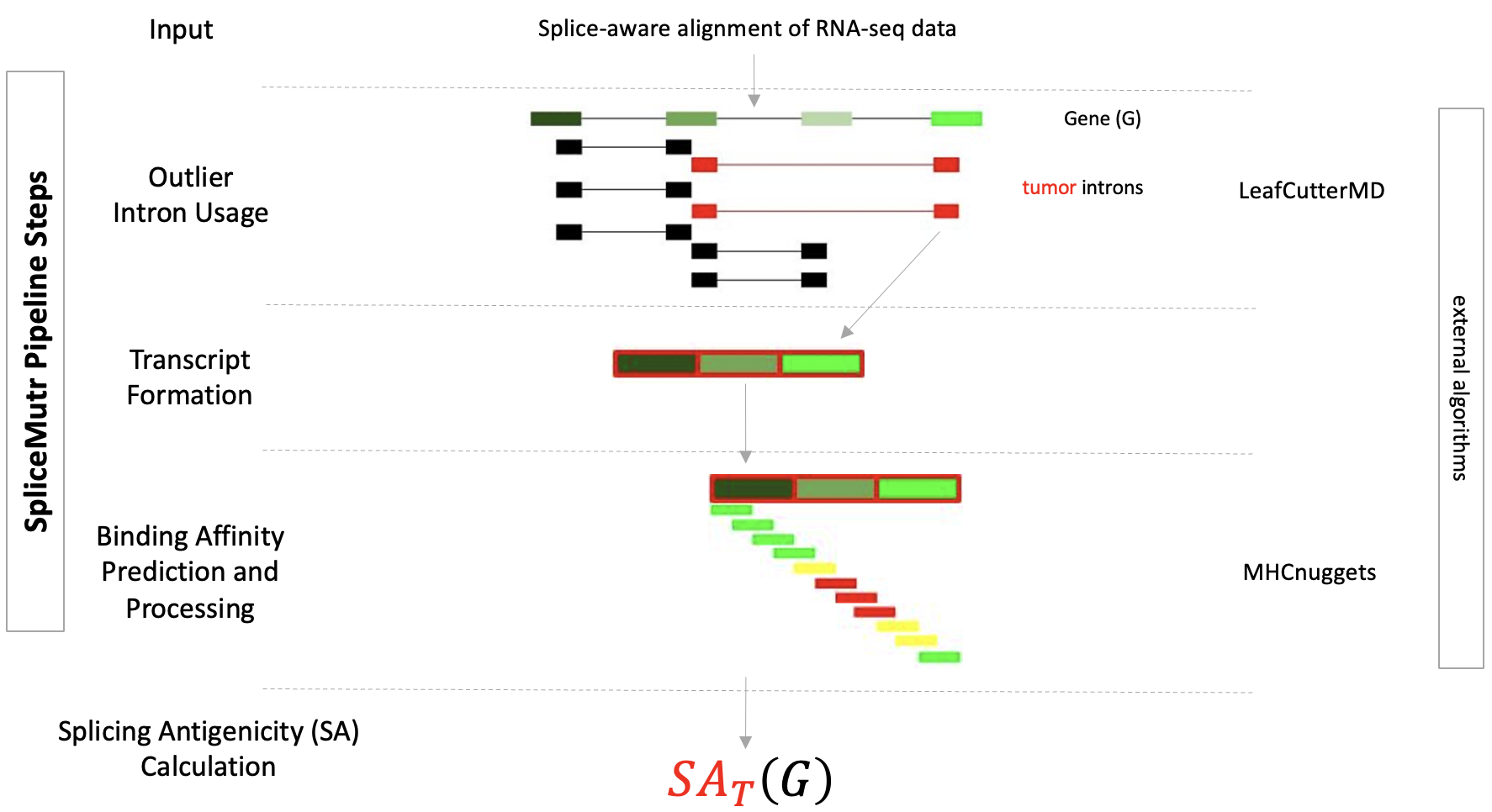


**Supplementary Figure S2. The SpliceMutr pipeline using LeafCutterMD outlier splicing vs LeafCutter differential splicing.** The SpliceMutr pipeline using LeafCutterMD uses RNA-seq data from one group of samples and calculates the splicing antigenicity for them. In this example, the alternative splicing analysis calcualates the splicing antigenicity for a set of tumor RNA-seq samples. The pipeline performs splicing-aware alignment and HLA genotyping. The splicing-aware alignment splice junction counts are then input into LeafCutterMD to evaluate outlier splice junction usage. The tumor-specific splice junctions undergo transcript formation, translation, and kmerization for MHCnuggets input through SpliceMutr, then are evaluated for genotype-specific MHC binders using MHCnuggets^22^. MHC binders associated with tumor-specific peptides are then used to calculate a per gene and sample splicing antigenicity (SA) metric for each sample. 𝑆𝐴_T_(𝐺) is calculated for each tumor sample.


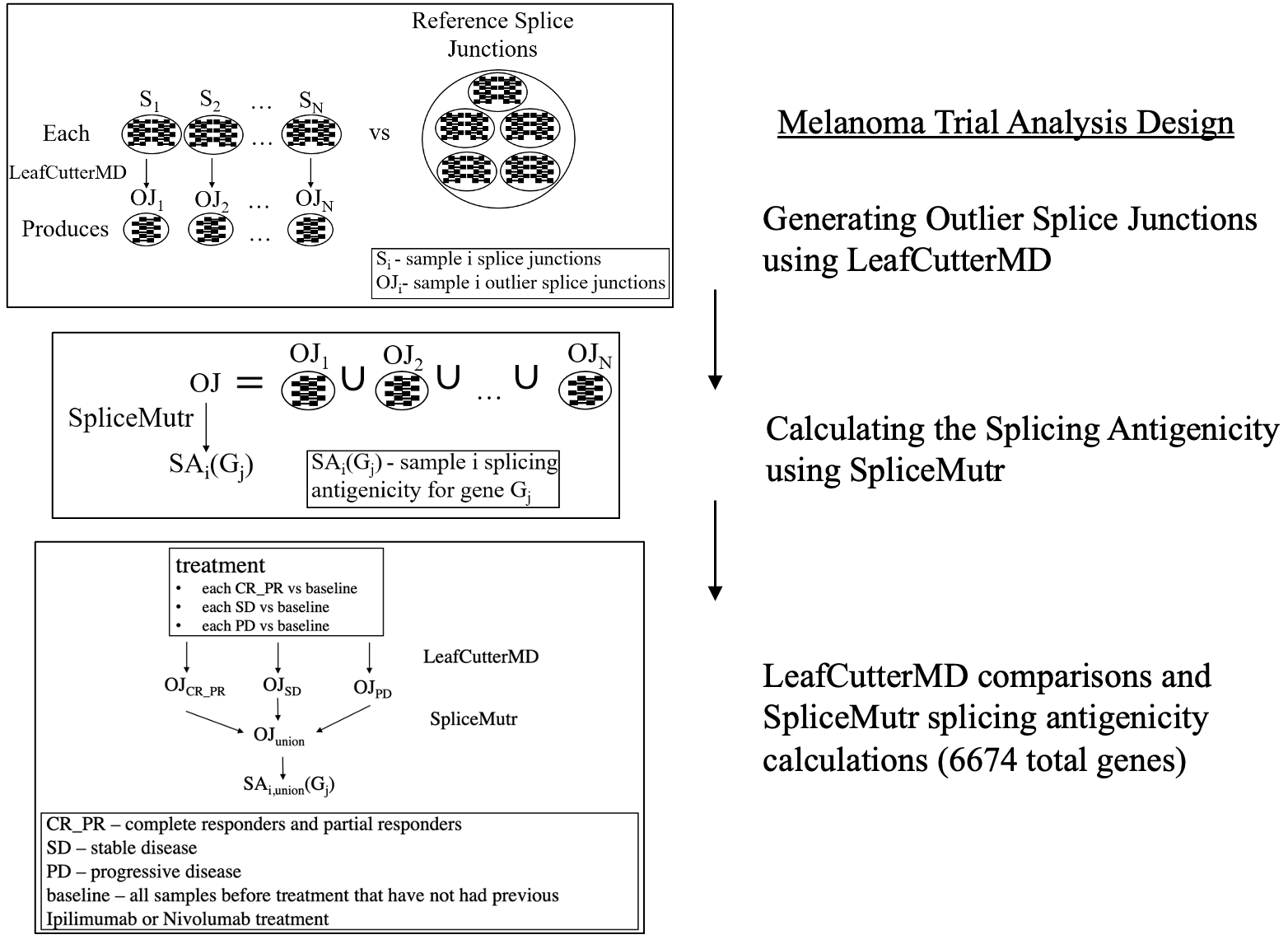


**Supplementary Figure S3 The melanoma cohort analysis design.** Outlier splice junctions are generated using LeafCutterMD. The set of splice junctions and their expression for all baseline samples is the reference set of splice junctions used to generate the outlier splice junctions per sample. The splicing antigenicity is calculated, per sample, using the union of the outlier splice junctions for a specific immune checkpoint inhibitor response group. There are three response groups, CR_PR (complete and partial responders), SD (stable disease), and PD (progressive disease). The union of the outlier junctions per response group are also used to calculate the splicing antigenicity to enable comparison of splicing antigenicity at the gene and splice-junction level across conditions.


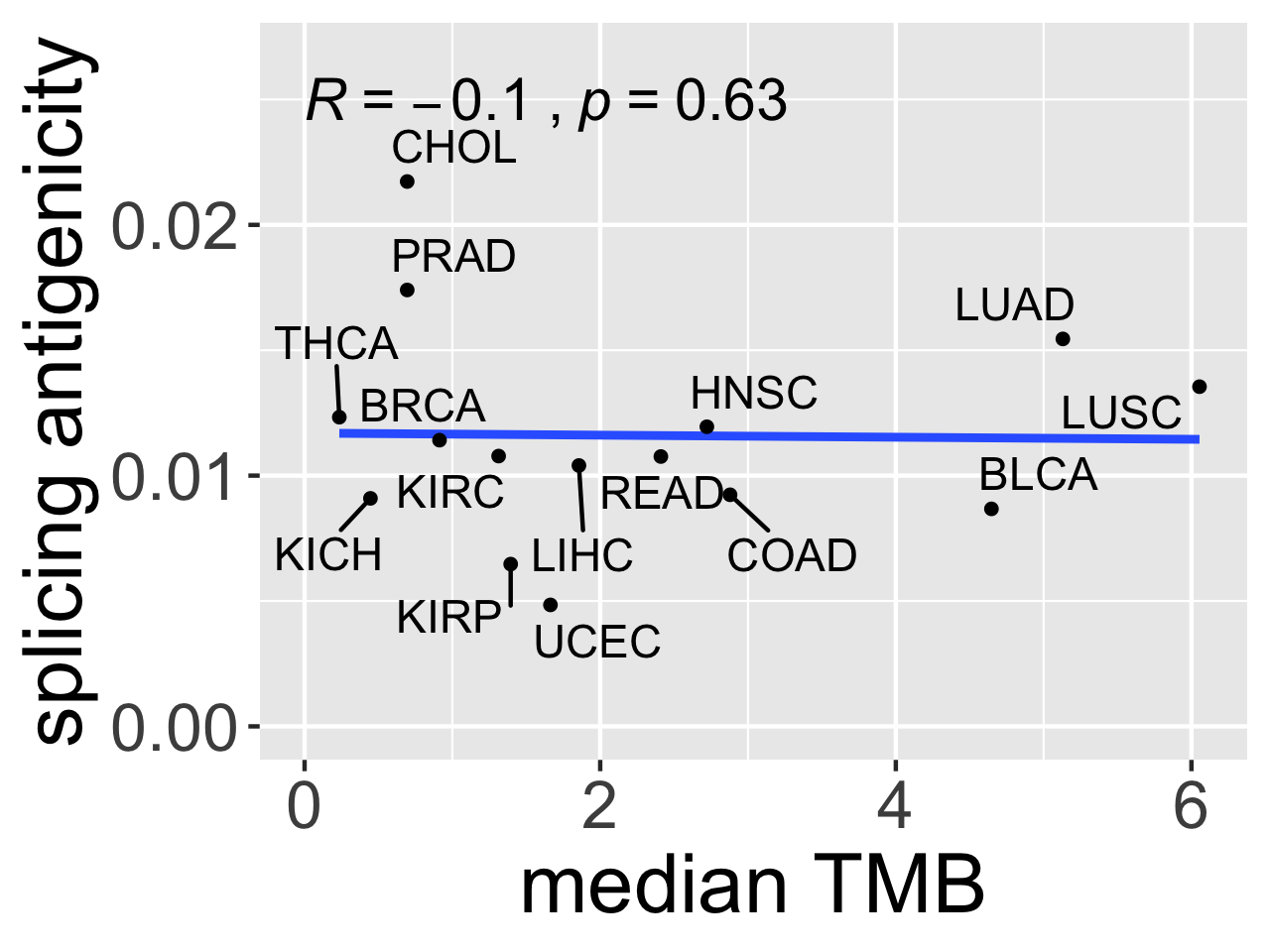


**Supplementary Figure S4. The median tumor mutational burden vs the splicing antigenicity.** The median TMB per cancer cohort vs the average splicing antigenicity per cancer cohort with correlation analysis performed using the Kendall Tau test.


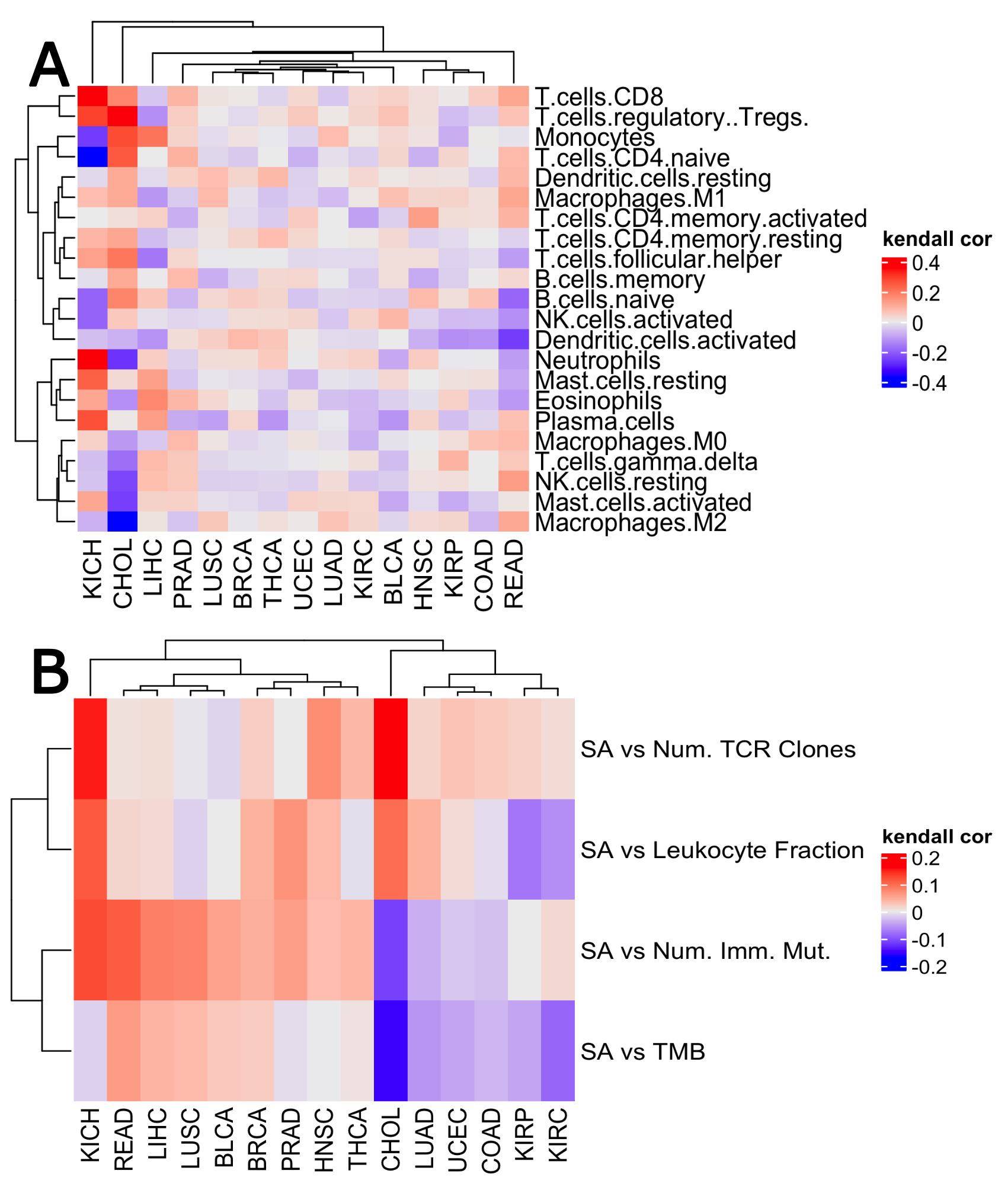


**Supplementary Figure S5.** (A) The splicing antigenicity correlated to CIBERSORT-calculated immune cell proportions per TCGA cancer subtype. (*: p-value BH < 0.05 and |tau| >= 0.1, Kendal Tau test). (B) The splicing antigenicity averaged across all genes per sample correlated to the TCR clonality, the number of immunogenic mutations, the leukocyte fraction, and the TMB per TCGA cancer subtype.


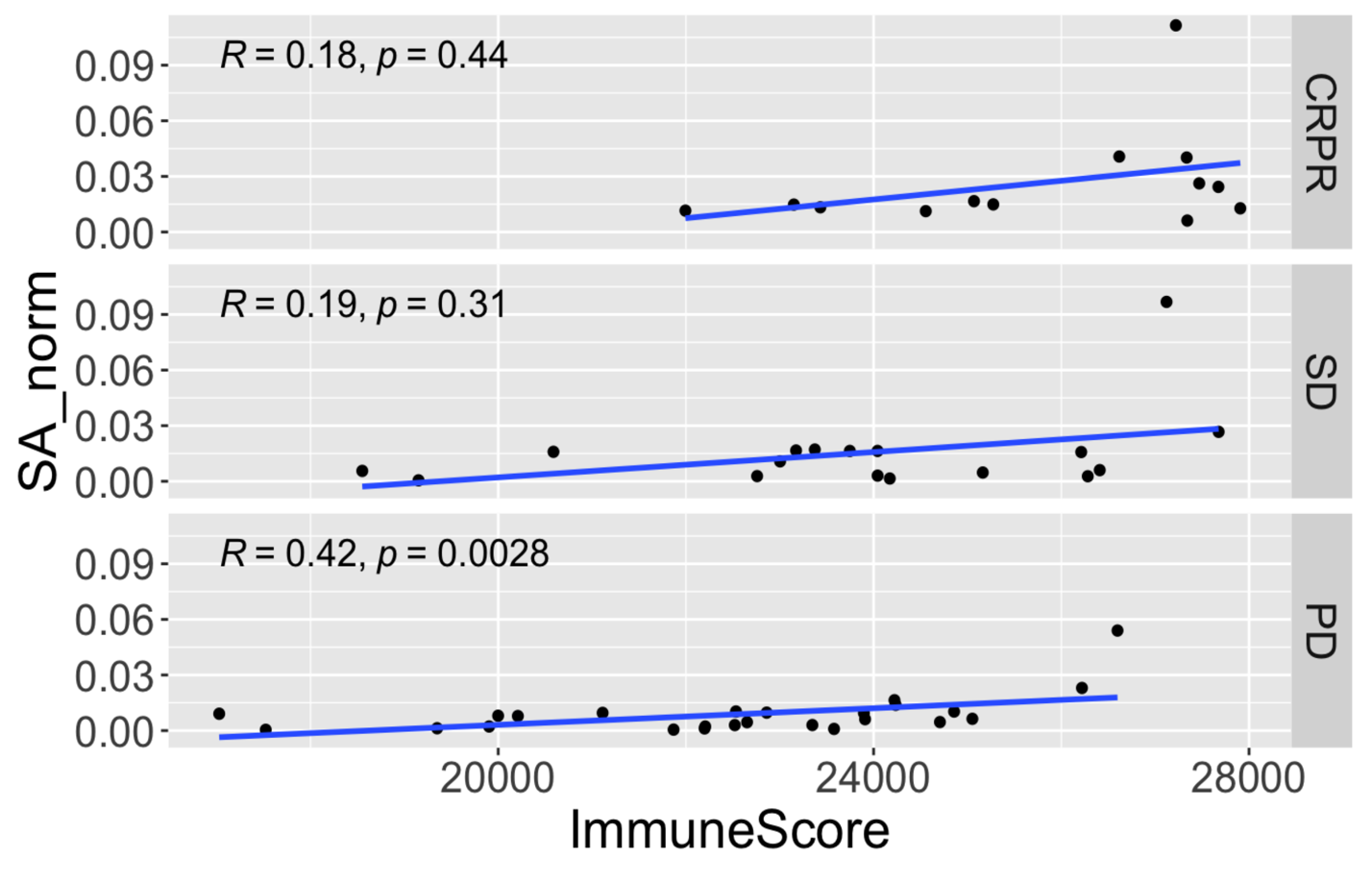


**Figure S6. The ESTIMATE immune score correlated with the splicing antigenicity.** The estimate immune score plotted against the normalized splicing antigenicity by response for the melanoma cohort. The kendal tau test statistic was used to test for significant correlation.


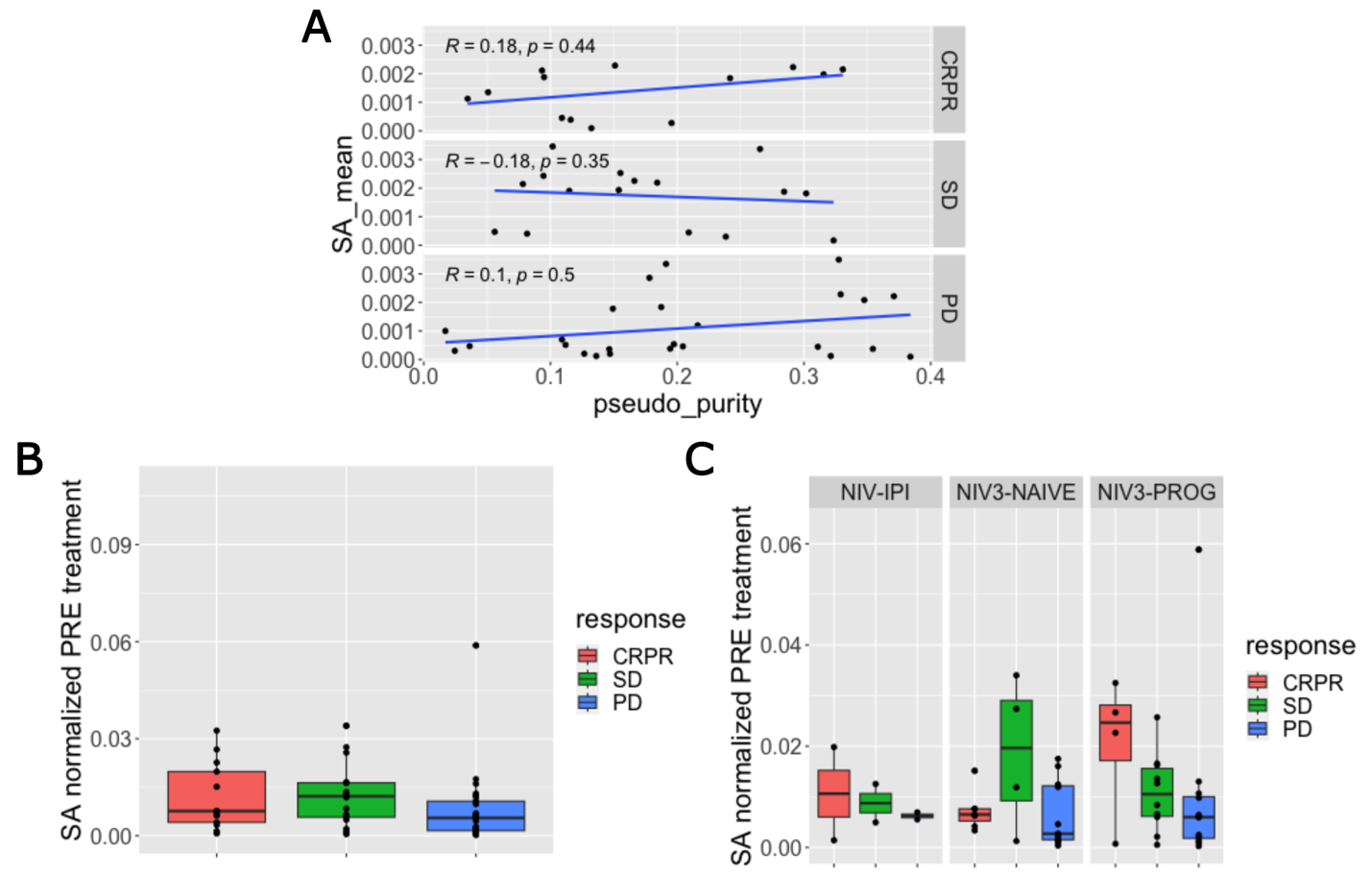


**Supplementary Figure S7. Per-patient splicing antigenicity of ICI-treated melanoma patients per treatment arm and response type before treatment.** (A) The per-patient pseudo purity compared to the mean splicing antigenicity averaged across genes per-response type for all treatment arms. Kendall Tau test.(B) The mean splicing antigenicity averaged across genes per patient and normalized by the pseudo purity, for each treatment arm. Wilcoxon test with false discovery rate adjustment and Cohens d. (C) The mean splicing antigenicity averaged across genes per patient and normalized by the pseudo purity, for all treatment arms combined. Wilcoxon test and Cohens d.


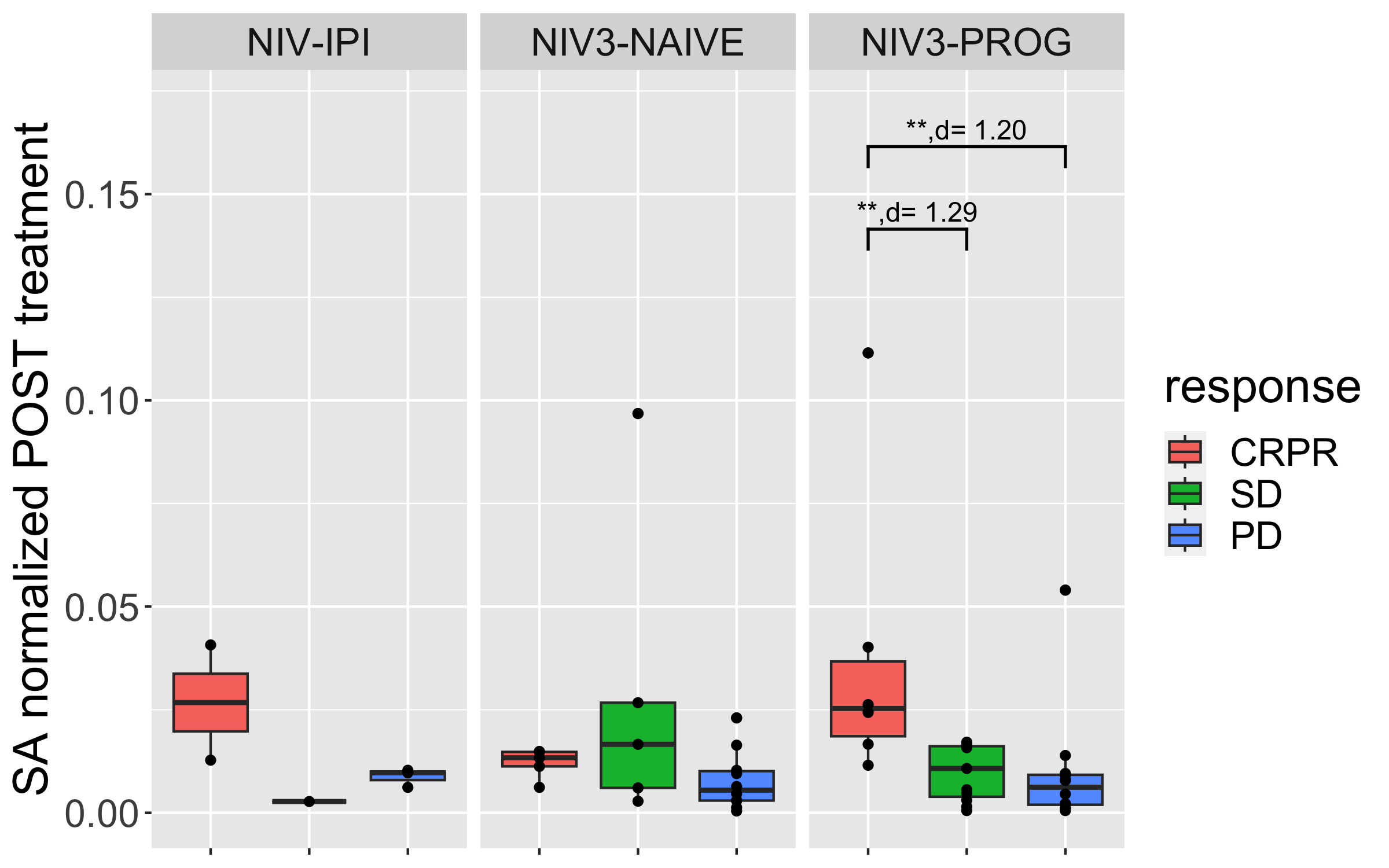


**Supplementary Figure S8. Per-patient splicing antigenicity of ICI-treated melanoma patients per treatment arm and response type after treatment.** The mean splicing antigenicity averaged across genes per patient and normalized by the pseudo purity, for each treatment arm. Wilcoxon test with false discovery rate adjustment and Cohen’s d.
